# Human Monocytotropic Ehrlichiosis, Missouri

**DOI:** 10.3201/eid0912.020733

**Published:** 2003-12

**Authors:** Juan P. Olano, Edwin Masters, Wayne Hogrefe, David H. Walker

**Affiliations:** * University of Texas Medical Branch, Galveston, Texas, USA; † Premier Family Physicians, Cape Girardeau, Missouri, USA; ‡ Focus Technologies, Cypress, California, USA

**Keywords:** *Ehrlichia chaffeensis*, *Anaplasma phagocytophilum*, *Ehrlichia ewingii*, human monocytotropic ehrlichiosis (HME), human anaplasmosis (formerly known as human granulocytotropic ehrlichiosis or HGE), polymerase chain reaction (PCR), Cape Girardeau

## Abstract

To determine the incidence, clinical and laboratory characteristics, and utility of molecular diagnosis of human monocytotropic ehrlichiosis (HME) in the primary care setting, we conducted a prospective study in an outpatient primary care clinic in Cape Girardeau, Missouri. One hundred and two patients with a history of fever for 3 days (>37.7°C), tick bite or exposure, and no other infectious disease diagnosis were enrolled between March 1997 and December 1999. HME was diagnosed in 29 patients by indirect immunofluorescent antibody assay and polymerase chain reaction (PCR). Clinical and laboratory manifestations included fever (100%), headache (72%), myalgia or arthralgia (69%), chills (45%), weakness (38%), nausea (38%), leukopenia (60%), thrombocytopenia (56%), and elevated aspartate aminotransferase level (52%). Hospitalization occurred in 41% of case-patients. PCR sensitivity was 56%; specificity, 100%. HME is a prevalent, potentially severe disease in southeastern Missouri that often requires hospitalization. Because clinical presentation of HME is nonspecific, PCR is useful in the diagnosis of acute HME.

Ehrlichioses were recognized as causing human infectious diseases relatively recently. Ehrlichiae, obligately-intracellular gram-negative bacteria, have evolved in close association with a vector arthropod and a zoonotic host and have been traditionally recognized as veterinary pathogens (*1*–*4*). In the United States, the first human case of ehrlichiosis was reported in 1987 (*4*). In 1991, the agent was isolated and recognized as a novel pathogen, *Ehrlichia chaffeensis* (*5*). By 1997, 742 cases in 47 states had been reported to the Centers for Disease Control and Prevention, most likely an underestimate of the true incidence (*6*). Passive reporting of cases has yielded the concept that even in the states with the most cases the incidence is low (e.g., 0.5 cases/100,000 persons in Arkansas). The clinical spectrum of human monocytotropic ehrlichiosis (HME) ranges from mild to a life-threatening multisystem disease (*7*–*11*) with a case-fatality rate of 2% to 3% and a duration of illness in the absence of antiehrlichial treatment averaging 3 weeks. The clinical manifestations are neither sensitive nor specific for the diagnosis of HME. Sequelae include asthenia that can continue months after recovery and an ill-defined immunosuppression that predisposes the patient to opportunistic infections. Conversely, *E. chaffeensis* can cause overwhelming infection in patients with AIDS or other immunosuppressive conditions (*12*–*14*).

The exploding population of the natural reservoir of *E. chaffeensis,* white-tailed deer, and the expansion of the range and population of the vector tick *Amblyomma americanum* are important ecologic factors in the continuing emergence of HME (*15*–*19*). Other tick-borne human granulocytotropic infections are caused by *Anaplasma phagocytophilum* and *E. ewingii*.

Although HME was described more than a decade ago, prospective studies are scarce (*8*,*20*–*22*). The present investigation describes the first office-based, prospective study of HME in the primary care setting, an investigation over a period of 3 years in southeast Missouri.

## Materials and Methods

### Epidemiologic and Clinical Data

The study area included Cape Girardeau and surrounding counties in southeast Missouri and southwestern Illinois. Approximately 100,000 persons were covered by the health services offered by the medical community. Patients were enrolled from March 1997 through December 1999. The clinical definition of a potential HME case-patient was a patient who had had fever (>37.7°C) for >3 days, possible tick bite or other tick exposure, and no other infectious disease diagnosis established. The patients were given two questionnaires, one during the acute phase of the disease and the second during the convalescent phase when the diagnosis of HME was confirmed by appropriate laboratory studies. A third questionnaire was given to the primary care provider. The information requested included the following: age, gender, occupation, tick exposure/bites, clinical signs and symptoms, duration of symptoms, occurrence and duration of hospitalization, antibiotic treatment, days of treatment until resolution of fever, and laboratory data. The protocol study was approved by the Institutional Review Board of the University of Texas Medical Branch.

### Statistical Analysis

All patient information and laboratory results were entered into Microsoft Excel worksheets (Microsoft Corp., Redmond, WA). Data were analyzed by using Sigma Stat Version 2.03 (SPSS Inc., Chicago, IL).

### Laboratory Case Definition Criteria

#### Definite and Probable HME Cases

A definite HME case was defined as follows: Patients who met the clinical definition and had one of the following conditions: a) serologic immunoglobulin (Ig ) G rise from <1:64 to >1:64 with a positive polymerase chain reaction (PCR) result, or b) IgG seroconversion (fourfold rise) to >1:128 without positive PCR or c) positive PCR results in two separate laboratories or for at least two target genes , or d) single serum Ig G titer of >1:256, or e) positive culture for *E. chaffeensis*.

A probable case of HME was defined as follows: Patients who met the clinical definition and had a) single IgG titers of 1:64 or 1:128, or b) positive PCR results in one laboratory for only one target gene.

### Processing of Blood Samples

The samples were collected in EDTA-containing tubes and shipped in wet ice overnight to the University of Texas Medical Branch in Galveston. The blood elements were separated by differential gradient centrifugation with Ficoll-Hypaque. The mononuclear band was harvested, washed twice in phosphate-buffered saline (PBS), and resuspended in 2 mL of PBS; 500 μL was then added to DH82, THP-1, and HL-60 cell cultures. The remaining 500 μL was saved for PCR analysis. Serum samples were received separately in red-topped tubes and kept at –20°C until antibody analysis was performed.

### Indirect Immunofluorescent Antibody Assays (IFA)

Serum specimens were screened at 1:64 dilution, according to a previously published protocol (*23*). Positive serum specimens were diluted serially in twofold increments to 1:4,096. The highest dilution with a 1+ intensity of fluorescent staining was considered the end-point titer. HL60 cells infected with *A. phagocytophilum* (Webster strain) were also used for IFA testing for HGE. The cut-off values for HGE testing were set at 1:80, and the samples were serially diluted to 1:1280.

### Preparation of DNA

DNA was extracted from the harvested mononuclear band by using the IsoQuick Extraction kit (ORCA Research, Bothell, WA) during the first year of the study and with the QIAgen DNA extraction kit (QIAgen, Santa Clarita, CA), according to the manufacturer’s instructions, during the remaining 2 years.

### PCR Reactions

#### 16S rRNA Subunit Gene

For the first-stage amplification of this gene, a 100-μL reaction mixture containing 10 μL of DNA template, 75 μL of sterile H_2_O, 10 μL of 10X PCR buffer (Boehringer Manheim, Indianapolis, IN), 1 μL of primers ECB and ECC (Table 1) at a final concentration of 1 μM each, 2 μL of deoxynucleotide triphosphates (final concentration, 200 μM), and 1 μL of *Taq* polymerase (Boehringer Manheim, Indianapolis, IN; final concentration 2.5 U). For nested PCR, 1 μL of each first-stage amplification reaction was amplified in a second 100-μL reaction tube after careful manipulation of the specimens in an AirClean 600 Workstation (AirClean Systems, Raleigh, NC) and aspiration of the PCR mixture with cotton-filled tips. The conditions were the same except for the use of species-specific primers for *E. chaffeensis*, HE1 and HE3 (Table 1).

**Table 1:** List of PCR primers used in this study for amplification of ehrlichial DNA sequences from blood specimens. Cape Girardeau, Missouri, 1997–1999

Target gene	Outside primer pair	Nested primer pair	Cycles: T° (time)* for outside primers	Cycles: T° (time)* for nested primers	
16S rRNA subunit gene. *E. chaffeensis*	ECB 5′CGTATTACCGCGGCTGCTGGA-3′ ECC 5′AGAACGAACGCTGGCGGCAGCC-3′	HE1 5′’CAATTGCTTATAACCTTTTCCTTATAAAT-3′ HE3 5′TATAGGTACCGTCATTATCTTCCCTAT-3'	94 (60) 45 (120) 72(60)	94 (60) 55 (120) 72(60)	
120-kDa protein gene *E. chaffeensis*	PXCF3 5′GAGAATTGATTGTGGAGTTGG-3′ PXAR4 5′ACATAACATTCCACTTTCAAA-3′	PXCF3b 5′-CAGCAAGAGCAAGAAGATGAC-3′ PXAR5 5′ATCT′	94(60) 48(120) 72(60)	94(60) 54(120) 72(60)	
*nadA* gene *E. chaffeensis*	ECHNADA1 5′-TCATTTCGTGCTTTCTTATTG-3′ PXCR6 5′-CAAACGCATATG TGGGCA-3′	NADPCR 5′ACGTCATTTGGCTCAGGA-3′ PXCR7 5′-TGTCGATCCAATGAAAT GAGC-3′	94(60) 48(120) 72(60)	94(60) 48(120) 72(60)	
16S rRNA subunit gene. *A. phagocytophilum*	PC5 5′-TACCTTGTTACGACTT-3′ Pomod 5′-AGAGTTTGATCCTGG-3′	GE9f 5′-AACGGATTATTCTTTATAGCTTGCT-3′ GE10r 5′-GGAGATTAGATCCTCTTAACGGAA-3′	94(60) 38(120) 72(60)	94(60) 60(120) 72(60)	

^a^ Temperature sequence: Denaturing, annealing and synthesis. Time given in seconds. All polymerase chain reactions (PCR) were performed for 35 cycles.

### 120-kD Protein Gene

The first-stage amplification reactions contained the same reagents as described above with the exception of *E. chaffeensis* species-specific primers for the 120-kD protein gene, PXCF3 and PXAR4. One μL was then amplified with nested primers for the 120-kD protein gene with primers PXCF3b and PXAR5 (Table 1).

### *nad* A Gene

The first-stage amplification was done under the same conditions as described for the other genes with primers ECHNADA1 and PXCR6. One μl was then amplified in a second 100-μL-reaction tube with nested primers specific for the *nad A* gene of *E. chaffeensis* NADPCR and PXCR7 (Table 1).

### 16S rRNA gene for HGE

The first-stage amplification reactions contained the same reagents as described above with the exception of the universal eubacterial primers for the 16S rRNA subunit gene, PC5 and Pomod. One microliter was then amplified with nested primers specific for *A. phagocytophilum*, GE9f, and GE10r (Table 1).

All reactions were performed in a PowerBlock II System (Ericomp Inc., San Diego, CA). The PCR products were then separated electrophoretically at 100V for 30 to 40 min in a 1.5% agarose gel and then stained with ethidium bromide. The gel was then examined under ultraviolet light.

### Sequence Analysis

The PCR products were purified by QIAquick, (QIAgen, Santa Clarita, CA). The nucleotide sequence was then determined by the dideoxynucleotide method of cycle sequencing with *Taq* polymerase (ABI Prism 377 DNA sequencer, Perkin-Elmer Corp., Foster City, CA). The sequencing reaction was carried out for each strand of DNA to avoid possible errors of incorporation of nucleotides by *Taq* polymerase. The sequences were analyzed by Genetics Computer Group, Wisconsin Package software and by Lasergene software (DNA Star, Inc., Madison, WI).

### Cultivation

Ehrlichial isolation was attempted by adding DH82, THP-1, and HL-60 cell lines as described above. The flasks were fed every 3–4 days as needed and kept for up to 60 days at 37°C and 5% CO_2_. Samples of the cell monolayers or suspensions were stained with DiffQuik weekly and evaluated for the presence of intracellular morulae. At the end of 60 days, and before discarding the flasks, DNA was extracted from the cell monolayers or flasks as described above. PCR was then performed with 16S rRNA ehrlichial primers that were used for the first-stage reactions described above.

## Results

### Demographic Findings

A total of 102 patients met the clinical definition criteria and were enrolled in the study during the 3-year period (three full tick seasons). HME was diagnosed in 29 patients on the basis of the defined criteria (case-patients, Table 2). Twenty-five of these cases were considered definite, and four were considered probable. Six cases were diagnosed in 1997, 14 in 1998, and nine in 1999. Seronegative patients from whom convalescent-phase serum samples were not obtained were excluded from the study as well as those who did not answer the questionnaires (53 patients). Of the 49 case-patients that were included in the final analysis, paired-serum samples were available in 33 cases. Twenty of these case-patients did not show seroconversion and therefore comprised the control group (noncase-patients). Twenty-one case-patients (72%) were male and eight case-patients (28%) were female. The age of the patients ranged from 15 to 78 years (mean: 48.2 years). The mean age for men was 48.8 years and for women, 46.1 years. Ages ranged from 15 to 70 years for men and 22 to 78 years for women. Twenty-three case-patients (79%) lived in a southeast Missouri county (Cape Girardeau, Bollinger, Scott, Stoddard, Phelps, and Perry) and six case-patients (21%) lived in a southwestern Illinois county (Union, Jackson and Johnson) (Figure). A tick bite was documented in 21 case-patients (72%), and tick exposure without a tick bite in 8 case-patients (28%). For all case-patients, tick attachment ranged from 24 to 72 hours, except for one case-patient who experienced tick attachment for 12 hours. The incubation period from observed tick bite until onset of illness ranged from 1 to 4 weeks. All cases occurred between April and mid-August. Two cases (7%) occurred in the month of August, five cases (17%) in May, three cases (10%) in both June and April, and 16 (55%) in July.

**Table 2:** Selected epidemiologic and laboratory results for 29 patients with human monocytotropic ehrlichiosis (HME). Cape Girardeau, 1997–1999

Patient no.	Age (y)	Sex	Y of diagnosis	PCR result^a^	IFA titer acute phase	IFA titer convalescent	WBC x 10^9^/L	Platelets x 10^9^/L	
1	44	M	1999	+	1:512	1:1024	1.9	90	
2	42	M	1999	+	1:256	1:512	3.5	114	
3	63	F	1999	–	1:1024	1:2048	6.4	83	
4	53	M	1999	+	Neg	1:512	4.5	180	
5	77	F	1999	–	1:1024	NA	3.5	44	
6	43	M	1999	+	1:512	NA	1.9	89	
7	48	M	1999	+	1:128	1:2048	4.0	NA	
8	30	M	1999	–	1:1024	NA	NA	NA	
9	28	F	1999	+	1:512	NA	5.4	NA	
10	22	F	1998	+	Neg	1:128	2.1	142	
11	59	M	1998	+	Neg	1:256	8.8	229	
12	67	M	1998	+	Neg	1:512	4.2	36	
13	78	F	1998	+	Neg	NA	NA	NA	
14	49	F	1998	+	1:4096	1:4096	2.6	271	
15	65	M	1998	–	1:256	1:1024	4.3	207	
16	26	M	1998	–	1:1024	NA	2.9	106	
17	44	F	1998	–	1:64	NA	10.0	397	
18	27	M	1998	–	1:64	NA	8.5	246	
19	24	F	1998	+	1:256	NA	2.4	69	
20	59	M	1998	–	1:1024	NA	4.9	102	
21	65	M	1998	–	1:256	NA	4.4	121	
22	52	M	1998	–	1:1024	1:1024	1.2	39	
23	54	M	1998	+	1:128	NA	NA	NA	
24	15	M	1997	–	Neg	1:64	6.4	308	
25	70	M	1997	+	Neg	NA	5.0	222	
26	47	F	1997	+	Neg	1:512	NA	NA	
27	31	M	1997	+	Neg	NA	3.5	56	
28	67	M	1997	–	1:2048	NA	5.2	208	
29	59	M	1997	–	1:4096	NA	6.9	166	

^a^Summary of all target genes used in the study: PCR, polymerase chain reaction; IFA, immunofluorescent assay; WBC, white blood cells; +, positive; –, negative; M, male; F, female; NA, not available.

**Figure:**
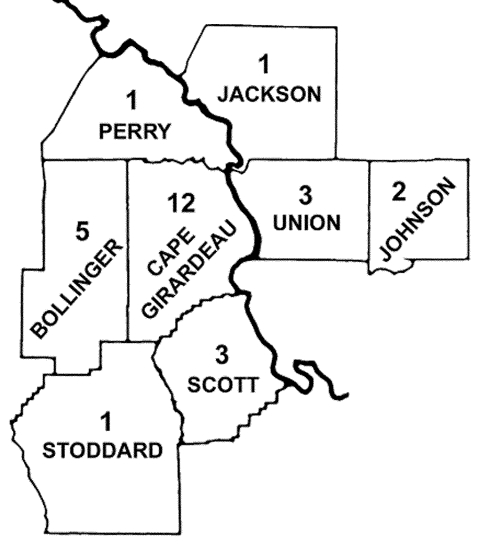
Counties in southeastern Missouri and southwestern Illinois in which cases of human monocytotropic ehrlichiosis (HME) were diagnosed from 1997 to 1999. Numbers represent HME cases in each county. A single case that occurred in Phelps County (south-central Missouri) is not shown

### Clinical and Laboratory Findings

A total of 29 cases were diagnosed with HME by IFA, PCR, or both. The clinical signs and symptoms associated included fever, headache, chills, weakness, nausea, vomiting, diarrhea, abdominal pain, dizziness, dyspnea, cough, sore throat, stiff neck and cutaneous rash (Table 3). Fever ranged from 37.9°C to 40.6°C (mean ± SD: 39.4°C ± 0.8). The most frequent symptoms besides fever were headache, myalgia or arthralgia, chills, weakness, and nausea. Coexisting conditions were found in three patients and included inflammatory bowel disease, adult onset diabetes mellitus, and coronary artery disease, status post coronary artery bypass grafting.

**Table 3:** Comparison of selected clinical features and laboratory data between patients with human monocytotropic ehrlichiosis (HME) (case-patient group) and noncase group (control group). Cape Girardeau, Missouri, 1997–1999

Clinical feature	HME case-patient group N (%)	Control group N (%)	p value
Fever	29(100)	20(100)	NA
Headache	21(72)	14(70)	0.89^a^
Dizziness	6(21)	2(7)	0.44^b^
Myalgia/arthralgia	20(69)	10(50)	0.29^a^
Chills	13(45)	7(35)	0.69^a^
Weakness	11(38)	3(15)	0.15^a^
Nausea	11(38)	3(15)	0.13^a^
Vomiting	2(7)	2(10)	1.00^b^
Diarrhea	3(10)	2(10)	1.00^b^
Abdominal pain	2(7)	1(5)	1.00^b^
Cough	7(24)	0(0)	0.03^b^
Sore throat	6(21)	0(0)	0.07^b^
Rash	6(21)	0(0)	0.07^b^
Stiff neck	6(21)	0(0)	0.07^b^
Confusion	2(7)	0(0)	0.50^b^
**Laboratory data**	Mean ± SD/median	Mean ± SD/median	p value
Age	48.6 ± 17.5	35.7 ± 19.9	0.02^c^
Leukocytes x 10^9^ cells/L	4.67	6.25	0.04^d^
Neutrophils x 109 cells/L	2645	3810	0.03^d^
Lymphocytes x 10^9^ cells/L	1677	1897	0.36^d^
Platelets x 10^9^ cells/L	172± 101.8	250.8 ± 137.5	0.06^c^
Aspartate aminotransferase (U/L)	63	32	0.84^d^

^a^Calculated by using Fisher exact test. ^b^Calculated by using chi-square test. ^c^Calculated by using t-test. ^d^Calculated by using Mann-Whitney rank sum test.

Hemoglobin values in all patients ranged from 102 to 169 gm/L (mean ± SD: 136 ±1.7 gm/L). Leukopenia (defined as leukocyte count [WBC] <4.5 cells x 10^9^/L) was present in 15 (60%). Of 25 cases in which WBC was analyzed, the overall range was from 1.2 to 10.0 x 10^9^ cells/L (mean ± SD: 4.6 ± 2.3 x 10^9^ cells/L). Of 23 patients in whom platelet counts were analyzed, the overall range was from 36 to 397 x 10^9^ cells /L (mean ± SD: 153.3 ± 95 x 10^9^ cells/L). Both thrombocytopenia and leukopenia were present in 11 patients (48%). Thrombocytopenia was observed in 13 (57%). Serum aspartate aminotransferase (AST) levels were determined in 21 patients and ranged from 18 to 538 U/L (mean ± SD: 124.1 ± 146.9 U/L). AST levels were elevated in 11 patients (52%). Serial blood cell counts were available in six patients, and all showed WBC returning to normal values from 7 to 21 days after the illness started. Lymphopenia was usually seen during the acute phase of the disease (both relative and absolute) and was replaced by relative and then absolute lymphocytosis, beginning at day 9 and occurring up until day 21 in some cases.

Altogether, 26 case-patients (90%) had serum antibodies detected by IFA. The three case-patients that were IFA negative were positive by PCR, and no convalescent-phase sample could be obtained from these patients. In fact, acute- and convalescent-phase samples were obtained in 13 patients from the case-patient group. Seroconversion (defined as a fourfold rise in end-point titers in acute- and convalescent-phase samples) was demonstrated in seven case-patients. The remaining case-patients, whose condition was diagnosed by IFA, had elevated titers in the acute- phase sample, and the titers rose slightly or remained stable in the convalescent-phase sample (Table 2). The geometric mean titer in the acute-phase samples was 512 and 633.7 in the convalescent-phase samples. The interval between acute- and convalescent-phase serum samples ranged from 2 to 8 weeks. In the acute-phase serum samples, nine patients (31%) had titers of <1:64, four (14%) had titers between 1:64 and 1:128, seven (24%) had titers between 1:256 and 1:512, and nine (31%) had titers >1:1024. Of the convalescent samples, two (15%) had titers between 1:64 and 1:128, five (39%) between 1:256 and 1:512, and six (46%) >1:1024. Cross-reactive antibodies against *A. phagocytophilum* were found in nine cases (31%), and all end-point titers were 1:160 or less. In all of these cases, the IFA end-point titers against *E. chaffeensis* were 1:512 or greater.

Ehrlichial DNA was amplified by PCR in 15 of the 29 confirmed and probable cases and in 14 of the 25 confirmed cases (sensitivity: 52% and 56%, respectively). Of the 14 HME patients who tested negative by PCR, 10 (71.4%) had IFA titers >1:256 (eight of these case-patients had titers >1:1024). Of the 15 cases diagnosed by PCR, ehrlichial DNA was amplified in nine cases from one target gene, in four from two target genes and in two from all three target genes used in the study. Twelve cases were diagnosed by both PCR and IFA. No ehrlichial DNA was amplified from acute-stage blood specimens of the 20 patients in the nonseroconversion control group (specificity >95%). PCR testing confirmed the infection in all but one of the patients who seroconverted (sensitivity: 84%).

The positive likelihood ratio for PCR was theoretically infinite since the specificity in our study was 100%. However, because of the relatively small number of cases, a specificity of >95% seems more adequate. In a hypothetical situation of one false-positive PCR result in 100 tests performed, the positive likelihood ratio would have been 56 and 84 for sensitivity values of 56% and 84%, respectively. The negative likelihood ratio was 0.44 for a sensitivity value of 56% and 0.16 for a value of 84%. The posttest probabilities for a positive PCR test were 97% and 96% for sensitivity values of 84% and 56%, respectively. The posttest probabilities of a negative PCR test were 4.3% and 11.1% for sensitivities of 84% and 56%, respectively. Posttest probabilities were calculated on the basis of the incidence of HME in the total population of the study (102 patients), that is, patients who met the case definition used in this study.

DNA sequencing analysis of PCR products was performed on samples from five patients that yielded PCR products for the 16S rRNA, *nadA* and 120-kDa protein genes. The sequences revealed greater than 99% homology with the published sequences of *E. chaffeensis* genes.

*Ehrlichia chaffeensis* was not cultivated from any of the blood samples that were shipped from Missouri to Texas.

Twelve (41%) of the HME patients required hospitalization: eight men and four women. Differences in age and laboratory data between hospitalized and nonhospitalized patients were not statistically significant, except for the degree of thrombocytopenia (Table 4).

**Table 4:** Association of selected demographic variables and laboratory data with severity of illness for 29 patients with human monocytotropic ehrlichiosis (HME), Cape Girardeau, Missouri, 1997–1999

Parameter	Nonhospitalized mean ± SD/Median	Hospitalized mean ± SD/median	p value	
Age (y)	45.8 ± 17.5	51.8 ± 19.1	0.41	
Leukocyte count, x 10^9^ L	5.1 ± 2.6	4.0 ± 1.7	0.21	
Platelets	192.4 ± 10^9^	117 ± 66	0.05	
Neutrophil counts, x 10^9^ ^a^	2,960	2,590	0.59	
Lymphocyte counts x 10^9^ L	1,948.5 ± 112.7	1,383.4 ± 1,167.7	0.26	
Aspartate aminotransferase, U/L^a^	89	49	0.96	

^a^Differences analyzed by Mann-Whitney rank sum test. All others analyzed by t-test.

All patients in whom HME was diagnosed were treated with doxycycline. Duration of treatment ranged from 2 to 4 weeks. Fever resolved within 24 hours in three patients (19%), within 48 hours in 10 patients (63%), and within 72 hours in three patients (19%).

Comparison of clinical parameters between HME case-patients and the control group showed no statistically significant differences between the two groups, except for the presence of cough in the HME case-patient group, illustrating again the nonspecific clinical presentation of this disease (Table 3). However, statistically significant differences between the two groups were observed for age, WBC count, and absolute neutrophil count, but not for platelet count, absolute lymphocyte count, or aspartate aminotransferase levels (Table 3).

## Discussion

HME is a prevalent disease in southeast Missouri, an area similar to most of the rural southeastern United States in terms of its white-tailed deer-lone star tick zoonotic cycle of *E. chaffeensis* and exposure to the bite of infected ticks. We enrolled 102 patients in the 3-year study, and 29 (28.4%) of patients had either definite or probable HME. For 1997, 1998, and 1999, the calculated incidence for HME was 2, 4.7, and 3 per 100,000 population, respectively (incidence calculations were based on the total population of all counties where the patients lived. Population figures were obtained from the U.S. Census Bureau Web site and are based on. the 2000 U.S. Census (URL: http://quickfacts.census.gov/qfd/states/29000.html). These incidence figures are higher than expected, even for an HME-endemic area such as Missouri. On the other hand, HME has probably been underestimated throughout the rural southeastern and south central states. In this particular disease-endemic area, our case-patients were identified mainly in one primary care-based physician’s office that cares for a population base of approximately 7,000 persons. Therefore, the real incidence of HME is likely higher in Cape Girardeau and surrounding counties than this overall study dictated. Physicians who diligently pursue the diagnosis are likely to be surprised by the frequency with which cases are identified. In fact, Carpenter et al. (*21*) reported a higher than expected incidence of HME in a prospective study performed in central North Carolina, an area well known for a high incidence of Rocky Mountain spotted fever.

Our clinical case definition was broad and tried to include all potential cases of HME in the disease-endemic area. Our laboratory criteria to diagnose HME in this study are patterned after those of the Council of State and Territorial Epidemiologists (CSTE), although our criteria are even more stringent regarding PCR interpretation (*24*). We required the amplification of ehrlichial DNA by two primer sets or confirmation of PCR results by two different laboratories. We also required positive serologic assays, along with the PCR results, to confirm a suspected case. Our aim was to avoid the inclusion of cases in which PCR might have amplified ehrlichial DNA nonspecifically. However, our specificity for PCR testing was 100%.

Our serologic criteria for laboratory diagnosis of HME are the same as those proposed by CSTE. For confirmation purposes, we considered end-point titers of 1:256 or greater as a criterion when only one serum sample was available for diagnosis. IFA seroconversion has been considered the standard criterion for the diagnosis of HME. However, samples with high end-point titers by IFA (>1:256) are highly suggestive of acute HME unless the patient is recovering from an acute infection and the titers are returning to normal levels. High end-point titers usually return to lower levels several months after the patient recovers clinically. In three of our case-patients, antibodies against *E. chaffeensis* were still detectable 8 to10 months after infection. In these cases, PCR or rising IFA titers would help solve the diagnostic dilemma. Frequently, diagnostic IFA end-point titers were lacking at the time of the patient’s first visit. In this series, 31% of acute-phase serum samples had a diagnostic titer. In addition, 45% of the samples that tested positive (>1:64) at the initial visit had titers <1:256. Therefore, convalescent-phase samples are highly desirable to confirm cases of HME reliably. Another important finding is the presence of cross-reacting antibodies against *A. phagocytophilum* in 31% of our patients. In these patients, the titers against *E. chaffeensis* were higher than the titers against *A. phagocytophilum*, and according to published criteria, these cases most likely represent HME instead of *A. phagocytophilum* infections (*25*). In addition, PCR testing did not detect *A. phagocytophilum* DNA in any of the patients’ blood samples. The proportion of patients with cross-reacting antibodies is higher than reported in other series, and at this time we do not know the reason for this finding (*6*,*26*).

*E. ewingii* infections likely occur in this patient population as well. The specificity of the PCR primer sequences ensure that none of the patients with infections diagnosed by PCR amplification had *E. ewingii* infection. In addition, we were able to test the 49 case-patients included in the final analysis of the study retrospectively. After the first reports of *E. ewingii* cases in humans in 1999, we retrieved DNA from our freezers from those 49 case-patients. *E. ewingii*–specific primers were used and no amplicons were obtained. The possibility that a serum specimen that contained antibodies stimulated by *E. ewingii* might have been labeled as indicating HME cannot be excluded, owing to cross-reactivity with *E. chaffeensis*.

The sensitivity of PCR was calculated on the basis of the total number of cases diagnosed by IFA. The relatively low sensitivity (56%) in our study when compared to that of Everett et al. (87%) and Standaert et al. (100%) is noteworthy. We do not have a clear explanation for this difference. However, in those series all patients in whom ehrlichial DNA was amplified from blood had low or negative IFA titers in the acute-phase serum sample, whereas in our series a substantial number of patients had acute-phase serum samples with high IFA end-point titers. This difference suggests that the ehrlichemia might be lower in cases where the immune response is well established. In fact, a t-test analysis of the geometric mean titer of PCR-positive versus PCR-negative persons yielded a statistically significant difference (p < <0.007), suggesting that seropositive patients are less likely to be PCR-positive. PCR sensitivity increased to 84% when only cases diagnosed by seroconversion were used to calculate it. The specificity of PCR was 100%.The positive and negative likelihood ratios and posttest probabilities based on sensitivity and specificity suggest that PCR is a useful tool for diagnosing HME in the early phase of the disease.

Our failure to isolate *E. chaffeensis* from these cases is most likely related to the delay in inoculating the blood samples of patients with HME into cell culture. The interval between blood sampling and inoculation may play a critical role when attempts to obtain isolates of *E. chaffeensis* are made (*22*).

The spectrum of illness in our study ranged from mild to life-threatening disease that required hospitalization and intensive care; 41% of the patients in our study were hospitalized. Since we detected cases based on a clinical definition that included fever for >3 days, we probably excluded the mildest cases of the disease in which a self-limited illness developed, which resolved spontaneously. In fact, asymptomatic seroconversion has been documented in soldiers who underwent field training and were exposed to ticks. However, whether the antigenic stimulation in those cases was actually triggered by *E. chaffeensis* or by some other antigenically related, less pathogenic bacterium, such as *E. ewingii* or the unnamed white-tailed deer *Anaplasma* species (both also associated with the lone star tick) is not known (*18*,*27*). The clinical syndrome of HME observed in this study is similar to that described in other series (*7*,*8*,*10*,*11*,*20*–*22*) in that it can be a serious illness that requires hospitalization in a large number of cases, even though the prospective, clinic-based nature of the study allowed more mild cases to be identified earlier in the course of illness.

Comparison of the case-patient group and the control group revealed the important difficulty in clinical diagnosis: few clinical symptoms differed between case-patients and non–case-patients. Even the signs and symptoms that showed some differences are nonspecific and can occur in other clinical conditions. The relatively high frequency of neurologic and respiratory signs is noteworthy, showing the potential severity of this disease. Among the few patients that underwent lumbar puncture in this study, the CSF showed pleocytosis with lymphocytic predominance (data not shown). Age, white blood cell counts, and absolute neutrophil counts were statistically significantly different between the HME and non-HME patients; thrombocytopenia was nearly statistically different (p = 0.06), pointing out again the importance of leukopenia and thrombocytopenia as diagnostic clues during the acute phase of the disease. The differences in age of the patients confirm once again that HME tends to affect older people more frequently than younger people (*E. chaffeensis* infection also may cause a milder illness in the young).

In summary, HME is an emerging tick-borne disease; its epidemiology and clinical spectrum are still being determined, and the incidence is higher than previously thought. The clinical diagnosis is challenging, and a high degree of suspicion is required to order specific diagnostic tests to confirm the diagnosis. PCR appears to be a useful diagnostic test during the early phase of this potentially life-threatening tick-borne zoonosis.
